# Supplementary material for: Bulk cell density and Wnt/TGFbeta signalling regulate mesendodermal patterning of human pluripotent stem cells
Source: Nat Commun. 2016 Dec 9;7:13602. doi: 10.1038/ncomms13602 (PMC5155150; doi:10.1038/ncomms13602)
Supplement: Supplementary Information — Supplementary Figures 1-7, Supplementary References. [file ncomms13602-s1.pdf]

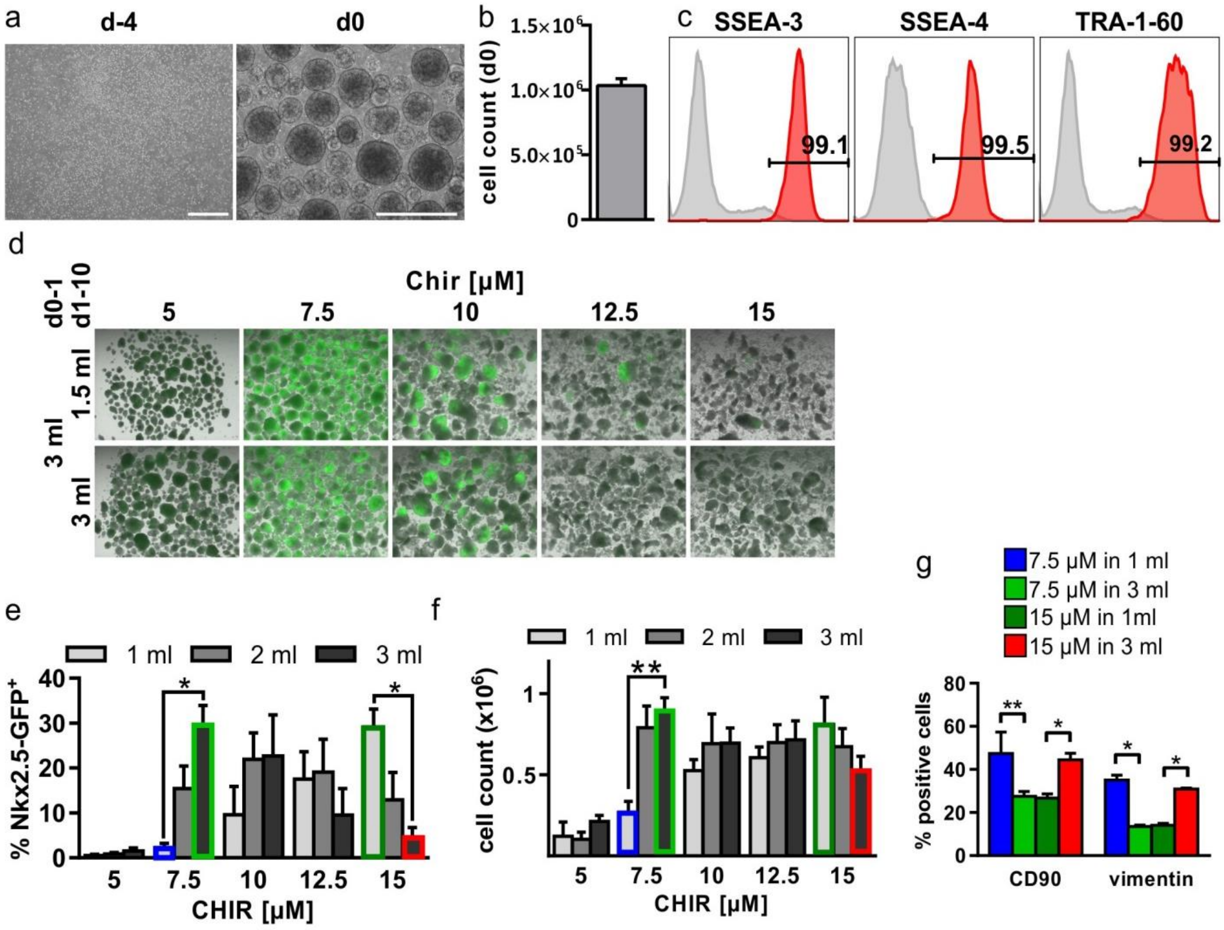

Supplementary Fig. 1, related to Fig. 1:Characterization of hPSC aggregates and additional data on the effect of the BCD during differentiation

(a) Representative images of single cell inoculated hPSCs on day -4 and resulting aggregates on day 0. scale bar = 500 µm **(b)** cell count of generated hPSC aggregates on day 0, n=9 of independent experiments **(c)** Representative flowcytometric analysis for pluripotency markers of hPSC aggregates on day 0. Isotype controls are shown in grey. **(d)** Modulation of the Volume (1.5 vs 3 mL) between day 1 and 10 did not affect the cardiac differentiation outcome. Representative images show the NKX2.5-GFP expression on day 10 following differentiation in 1.5 and 3 ml medium volumes between day 1-10. All conditions were kept in 3 ml medium during the first 24h at 7.5 µM CHIR. **(e,f)** The graphs show the results of the initial experiments that where conducted to assess the robustness of the differentiation outcome. Analysis shows levels of NKX2.5-GFP$^+$ on day 10 applying IWR1 instead of IWP2 on day 3 and respective total cell counts. The cornerstone conditions are marked in the respective color code. n=3-5 independent experiments; *P<0.05, *P<0.01, evaluated by two-way ANOVA followed by Bonferroni's post-test. **(g)** Flowcytometric analysis on day 10 indicates increased levels of fibroblast-like cells marked by CD90 and Vimentin in non-cardiac conditions (7.5/1 and 15/3). n=2-3. *P<0.05 and **P<0.01 evaluated by two-way ANOVA followed by Bonferroni's post-test. All bar graphs in this figure represent mean±s.e.m..

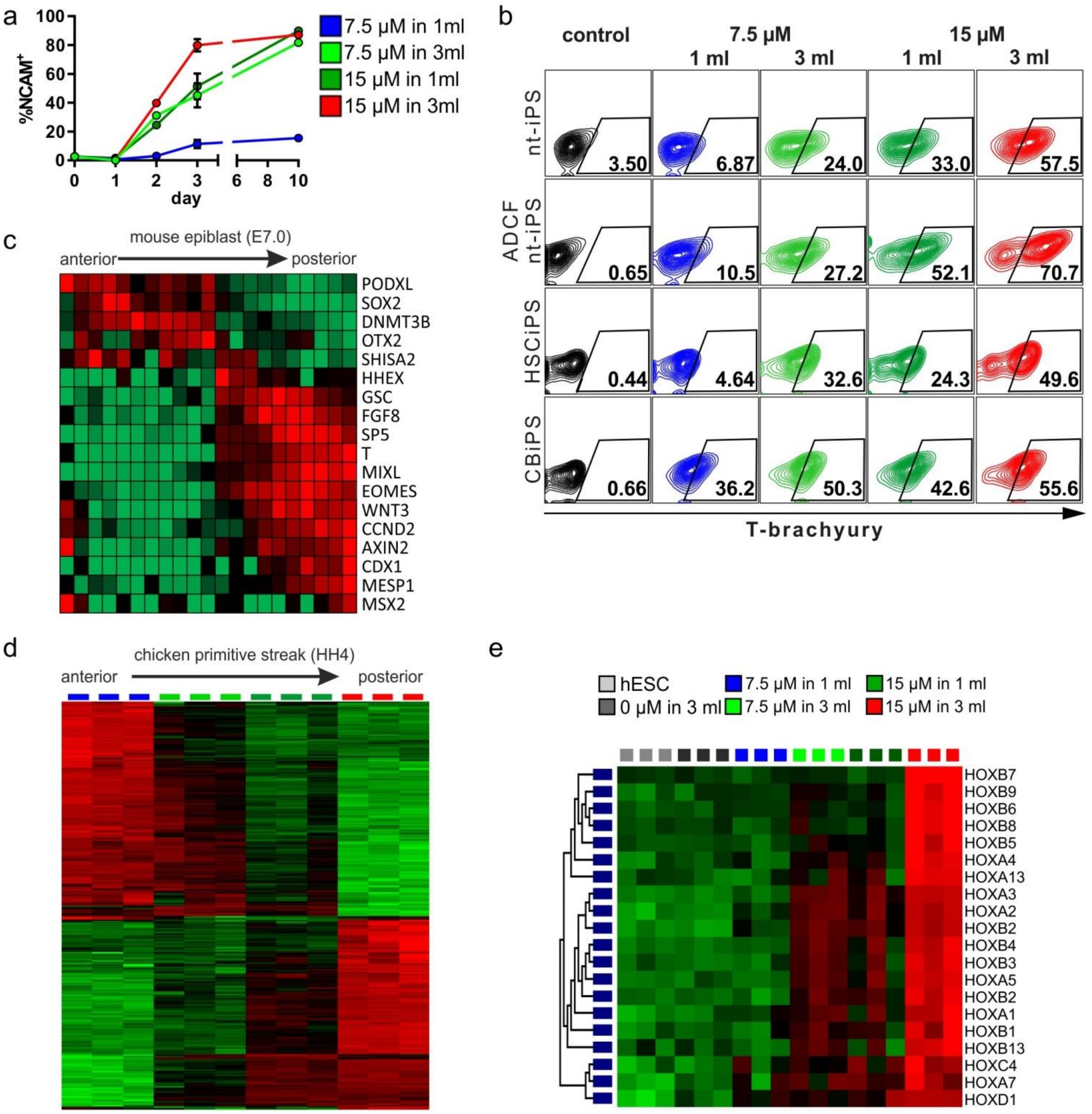

Supplementary Fig. 2, related to Fig. 2:Characterization and comparison of differentiation in other cell lines and species.
(a) NCAM$^+$ in the course of differentiation in the 4 cornerstone conditions (d0-d10). n=1 for d2, n=2 for d1 and 10, n=3 for d0 and n=8 for d3. Data shown as mean±s.e.m.. **(b)** T-brachyury expression determined by flow cytometry for 4 different hiPS cell lines after 24h of CHIR treatment in respective volumes and concentrations. Respective iPSC lines comprise lines of independent cell sources and reprogramming methods as described in the supplementary methods. **(c)** Heatmap showing the spatial resolution of key pluripotency, primitive streak as well as markers for early mesendoderm along the anteroposterior axis in the mouse epiblast (E7.0; www.itranscriptome.org) reveals no exclusive expression of genes in the mid-primitive streak.[1] **(d)** Gene expression pattern of the chicken primitive streak (PS at Hamburger and Hamilton stage 4 further dissected into 4 quarter along the anteroposterior axis; accession number GSE22230) reveals no exclusive expression of genes in the mid-streak region, but gradients along the anteroposterior axis.[2] **(e)** Heatmap of differentially regulated HOX gene obtained after 24h of differentiation at different CHIR and Volumes ($p<0.001$). red=high expression; green=low expression.

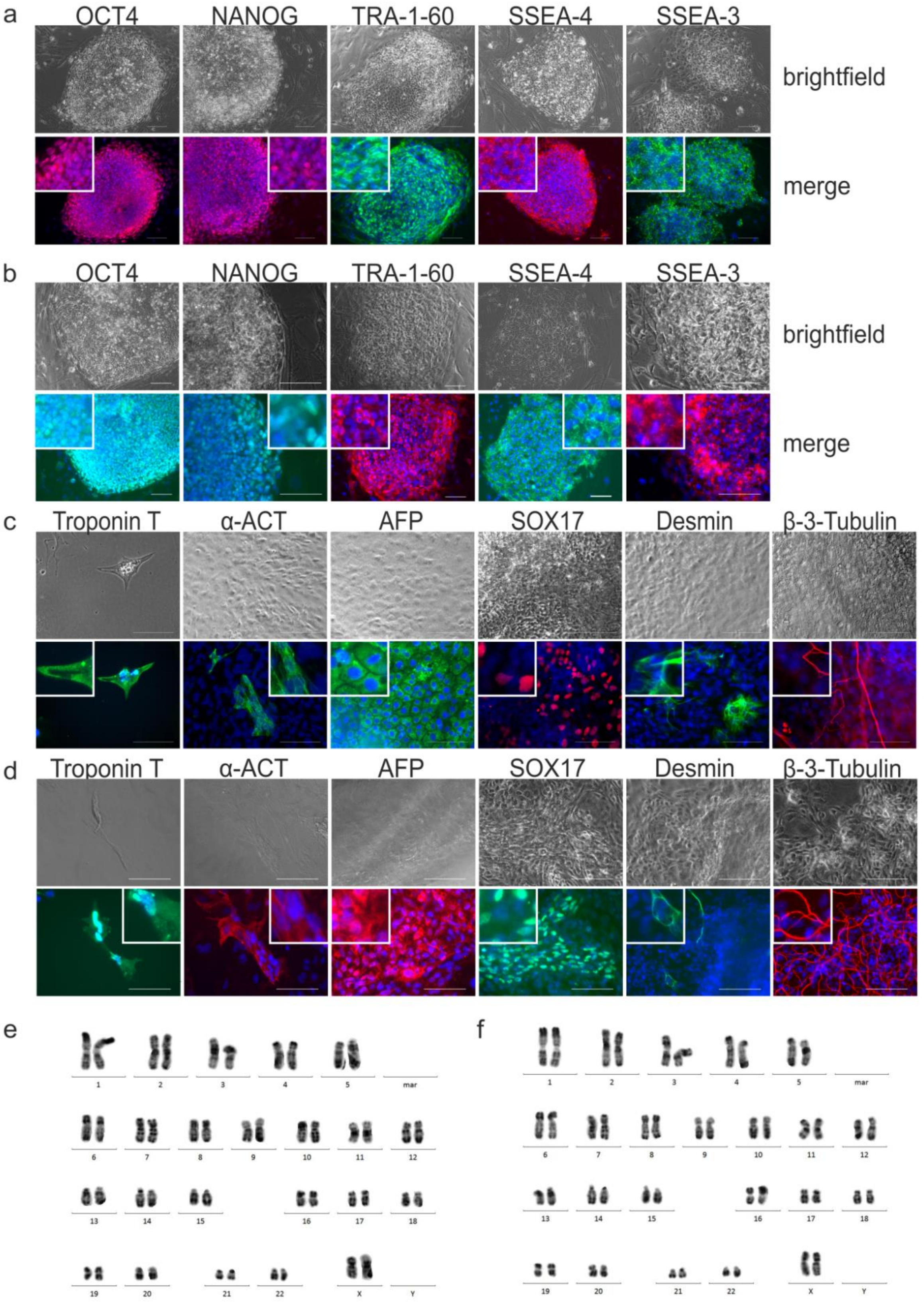

Supplementary Fig. 3, related to Fig. 2: Characterization of non-transgenic iPS cell lines
Images show representative colonies stained for the pluripotency markers OCT3, Nanog, TRA-1-60, SSEA-4, SSEA-3 from the non-transgenic hHSC_F1488_SeV-iPS2 (nt-iPS) cell line **(a)** and the animal-derived component free, non-transgenic hHSC_Iso4_ADCF_SeV-iPS2 (ACF nt-iPS) cell line **(b)**. Staining with markers of the three germlayers on day 21 after induction of spontaneous differentiation confirmed pluripotency for both, the nt-iPS **(c)** and the ACF nt-iPS cell line **(d)**. Both cell lines showed a normal 46XX karyotype **(e,f)**.

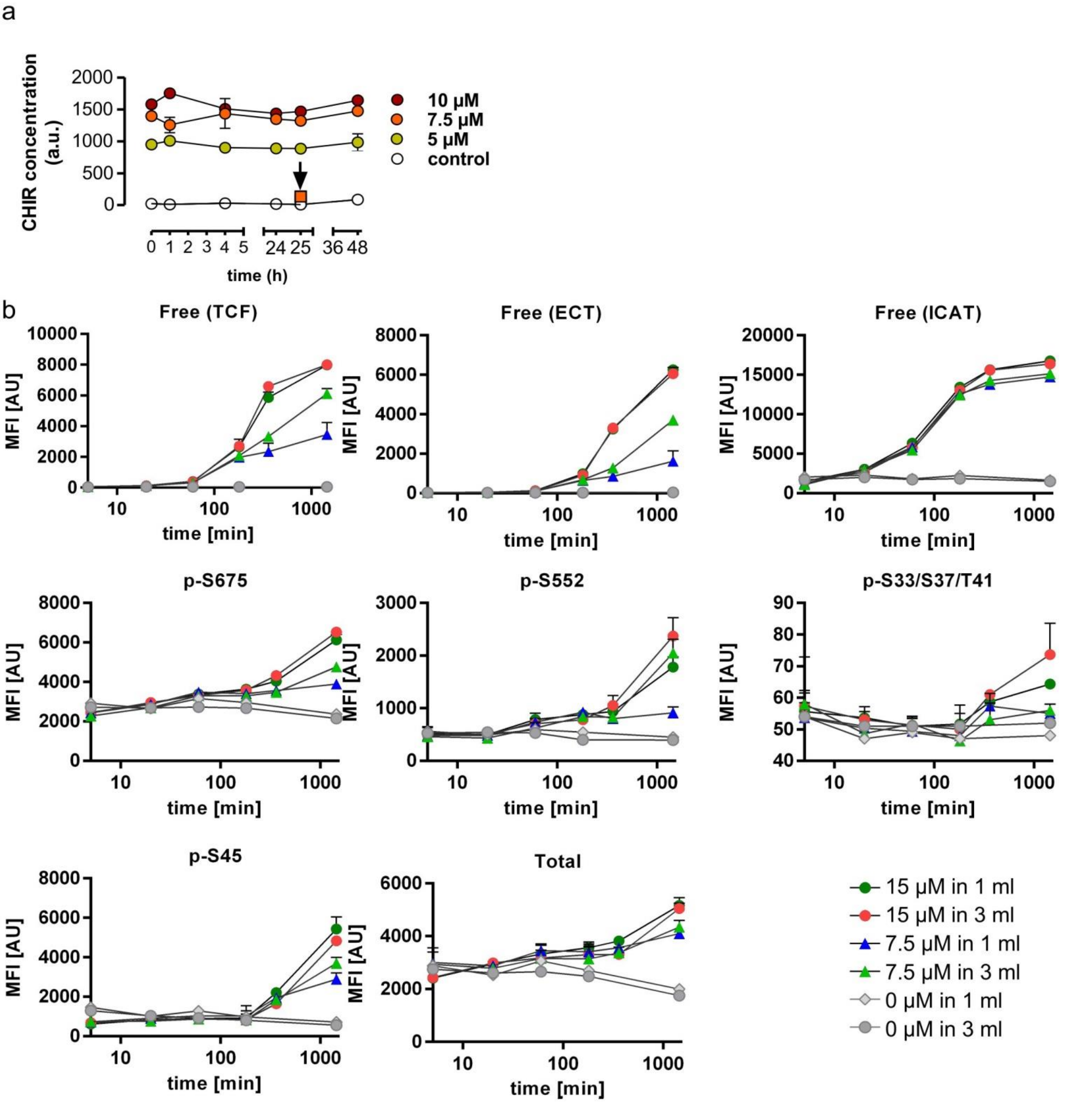

Supplementary Fig. 4, related to Fig. 3: Time-course analysis of CHIR concentration and WNT pathway activity

(a) CHIR concentration in the medium determined by mass spectrometry. The arrow indicates the concentration at 7.5 µM following a medium exchange after 24h (corresponding to 1440 min). n=2-4 biological replicates. Data shown as mean±s.e.m.. **(b)** Analysis of β-catenin signaling activity and phosphorylation during the first 24h of differentiation. n=3 of biological replicates. Data shown as mean±s.e.m..

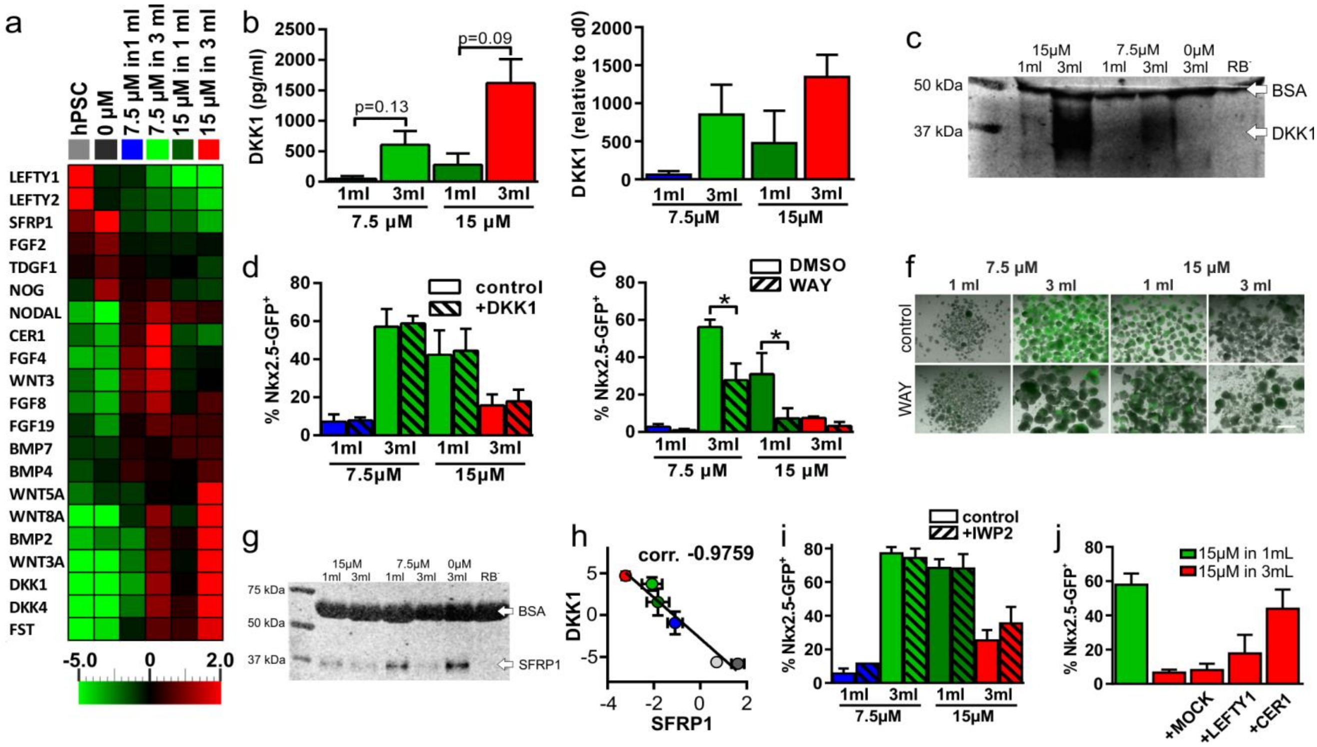

Supplementary Fig. 5, related to Fig. 4: Differential accumulation of secreted factors in the cornerstone conditions.

(a) Heatmap showing differentially expressed growth factors after 24h of differentiation in the 4 conditions and controls ($p<0.05$). **(b)** Concentrations of DKK1 in the supernatant after 24h of CHIR treatment detected by quantitative protein array (left) and corresponding gene expression pattern (right). **(c)** Representative Western blot for DKK1 of the 4 cornerstone conditions and controls from harvested supernatant after 24h confirms the data obtained in the microarray and proteinarray. The calculated molecular weight of DKK1 is ~29kDa, but the actual DKK1 band appears at 35-45kD suggesting pronounced glycosylation in line with previous work[3]. **(d, e, f)** NKX2.5-GFP$^+$ levels on day 10 following concomitant addition of DKK1 **(d)** and concomitant inhibition of DKK1 by WAY262611 **(e)** during the first 24h and respective images thereof **(f)**. n=3 independent experiments. *P<0.05, evaluated by one-way ANOVA with post-hoc Bonferroni analysis. **(g)** Representative Western blot for SFRP1 in the 4 cornerstone conditions and controls of the harvested medium after 24h. **(h)** Correlation in gene expression of DKK1 and SFRP1 in the 4 conditions and controls. (i) NKX2.5-GFP$^+$ levels on day 10 following concomitant addition of IWP2. n=2 independent experiments **(j)** Addition of enriched supernatant from HEK cells transfected with plasmids expressing LEFTY1 (Hölzel Diagnostika HG11877-UT) and CER1 (Hölzel Diagnostika HG10820-UT) partially rescued the cardiac differentiation compared to untreated controls and supernatant from mock-transfected controls (n=2 of independent experiments). All bar graphs in this figure represent mean±s.e.m..

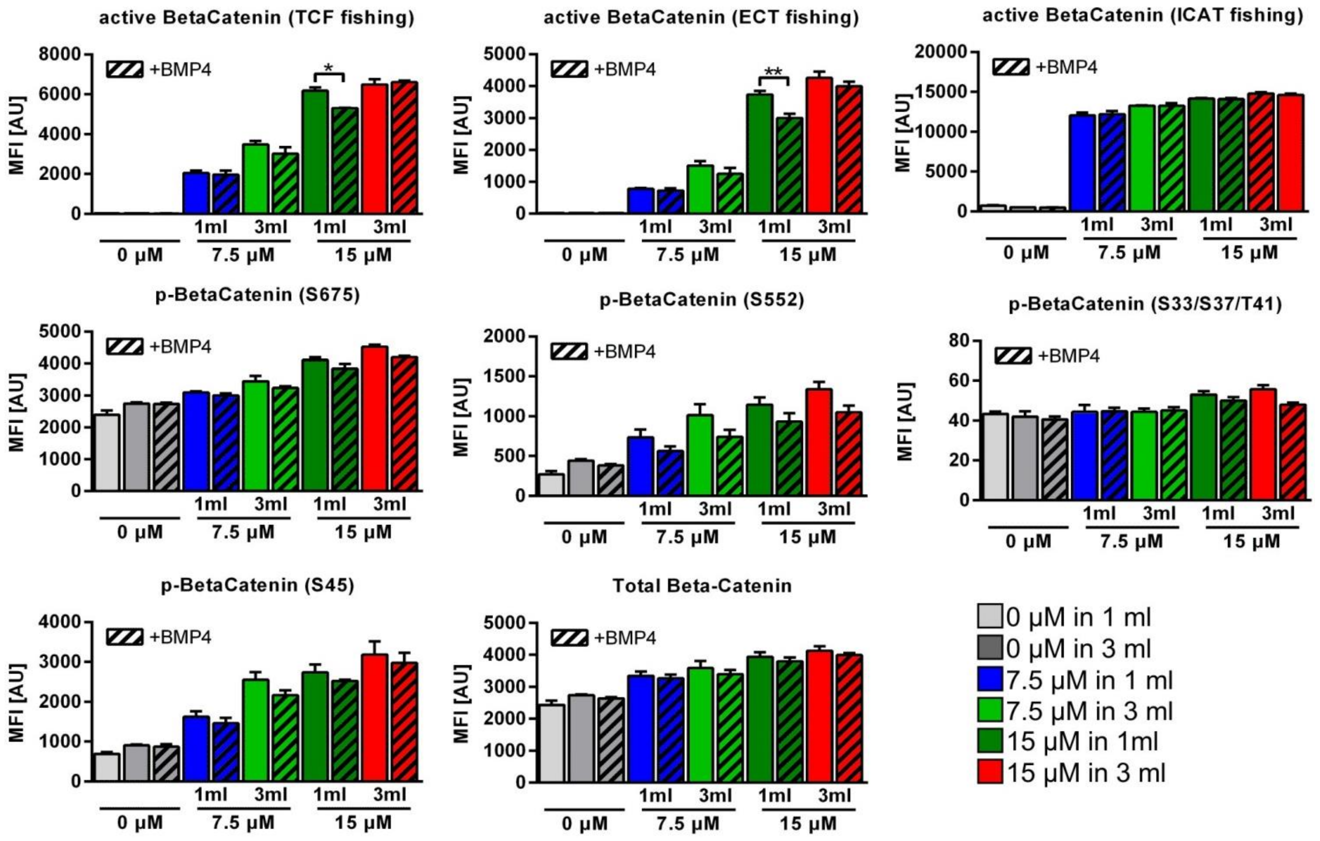

Supplementary Fig. 6, related to Fig. 3 and 4: Effect of BMP4 on canonical WNT pathway activity

Analysis of β-catenin signaling activity and phosphorylation on day 1 of differentiation with concomitant addition of BMP4. n=3 of biological replicates. *P<0.05, **P<0.01, evaluated for each target by one-way ANOVA with post-hoc Bonferroni analysis. All bars shown in this figure represent mean±s.e.m..

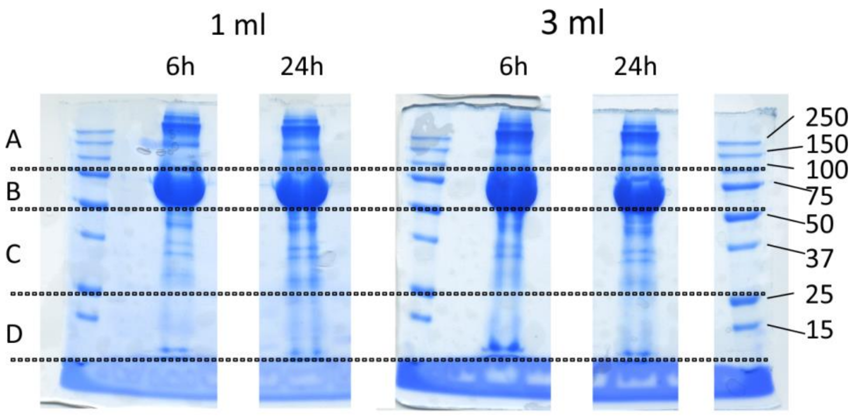

Supplementary Fig. 7: Gel of coomassie-stained proteins precipitated from culture supernatants, which was applied for excision of protein bands and subsequent mass spectrometric analysis.
Lanes were excised as indicated by dashed lines, further dissected and digested using trypsin. Lane B was excluded from the analysis due to the high BSA content.

Supplementary References:

1. Peng, G. *et al.* Spatial Transcriptome for the Molecular Annotation of Lineage Fates and Cell Identity in Mid-gastrula Mouse Embryo. *Developmental cell* **36**, 681-697 (2016).
2. Alev, C. *et al.* Transcriptomic landscape of the primitive streak. *Development* **137**, 2863-2874 (2010).
3. Niehrs, C. Function and biological roles of the Dickkopf family of Wnt modulators. *Oncogene* **25**, 7469-7481 (2006).
